# Jagged1 contributes to the drug resistance of Jurkat cells in contact with human umbilical cord-derived mesenchymal stem cells

**DOI:** 10.3892/ol.2013.1523

**Published:** 2013-08-12

**Authors:** YIN YUAN, XIN LU, XUAN CHEN, HONGWEI SHAO, SHULIN HUANG

**Affiliations:** 1 Southern Medical University, Guangzhou, Guangdong 510515, P.R. China; 2 Guangdong Provincial Key Laboratory of Biotechnology Candidate Drug Research, School of Life Science and Biopharmaceutics, Guangdong Pharmaceutical University, Guangzhou, Guangdong 510006, P.R. China; 3 School of Life Science, South China Normal University, Guangzhou, Guangdong 510631, P.R. China

**Keywords:** Jagged1, T-cell acute lymphoblastic leukemia, apoptosis, mesenchymal stem cells

## Abstract

Notch signaling, which is driven by the Notch1 receptor, plays an essential role in the pathogenesis and stroma-mediated drug resistance of T-cell acute lymphoblastic leukemia (T-ALL). However, little is known about the roles of Notch ligands in the survival or drug resistance of T-ALL cells. In the present study, isolated mesenchymal stem cells (MSCs) from human umbilical cord (hUC) samples, termed hUC-MSCs, were used as stromal cells for the Jurkat T-ALL cell line. The role of the Notch ligand, Jagged1, was assessed in the survival of Jurkat T-ALL cells using this co-culture system. hUC-MSCs and Jurkat cells were observed to express Jagged1. Furthermore, co-culture with hUC-MSCs led to a significant upregulation of Jagged1 and a more significant overexpression of its receptor, Notch1, in the Jurkat cells, indicating that the receptor and ligand pair may play a role in the reciprocal or autonomous activation of the Notch pathway. In addition, a higher level of CD28 expression was observed in the Jurkat cells that were co-cultured with hUC-MSCs. Blocking Jagged1 expression using neutralizing antibodies restored drug-induced apoptosis in the Jurkat cells that were co-cultured with hUC-MSCs, and also increased the drug sensitivity of the Jurkat cells that were cultured alone. By contrast, direct incubation with exogenously recombinant Jagged1 produced the same protective effects in Jurkat cells as those induced by hUC-MSCs. These results indicate a significant role for Jagged1 in hUC-MSC-induced survival and the self-maintenance of the Jurkat T-ALL cell line, making it a potential target for the treatment of human T-ALL.

## Introduction

T-cell acute lymphoblastic leukemia (T-ALL) is an aggressive malignant disease induced by the malignant transformation of T-cell precursors. T-ALL accounts for 10–15% of all leukemias in children and adolescents (1). The molecular mechanisms underpinning T-ALL are likely to be complex (2). A series of studies have demonstrated that the abnormal activation of the Notch1 signaling pathway plays a significant role in the pathogenesis of T-ALL (3,4).

The Notch1 gene encodes a single-pass heterodimeric transmembrane receptor, which has a fundamental function in the development of normal T cells (5). Normally, the activation of Notch signaling is triggered by Notch receptor-ligand interactions. The direct binding of a ligand from a signaling cell to a Notch receptor on the membrane of a receiving cell initiates two successive proteolytic cleavages by the TNF-α-converting enzyme (TACE) and the γ-secretase/presenilin complex. The proteolytic cleavage ultimately results in the release of the Notch intracellular domain (NICD), which translocates into the cell nucleus and interacts with the recombination signal binding protein Jκ (RBP-J). The NICD/RBP-J complex transactivates downstream target genes, including the hairy/enhancer-of-split 1 (Hes-1) gene (6). However, in T-ALL patients, mutations in the Notch1 gene are common and may lead to aberrant activation of Notch signaling that is independent of ligand binding (3). By contrast, the Notch1 proteins in the T-ALL cells also serve as surface receptors that may be triggered by Notch ligands that are expressed by specific cell types, including bone marrow stromal cells. Increasing evidence has suggested that the interaction between tumor cells and the stromal microenvironment results in the resistance to chemotherapy in leukemia and myeloma (7,8). Notch signaling has been shown to be one of the molecular mechanisms involved. It has been shown that the signaling driven by Notch1 may inhibit apoptosis in developing thymocytes, mature T cells and T-ALL cells (9–11).

In contrast to the roles of the Notch1 receptor, the roles for Notch ligands in T-ALL biology are less clear. The known Notch ligands in mammals include Jagged1 and 2, and Delta-like (DLL)-1, 3 and 4 (6). The actions of these ligands differ in the initiation of Notch signaling and may result in a diverse or opposed biological outcome (12). The present study assessed the role of Jagged1 in the survival of Jurkat T-ALL cells when exposed to a cytotoxic drug, with or without stromal support.

Stromal cells derive from their mesodermal precursors, mesenchymal stem cells (MSCs), which are non-hematopoietic progenitor cells that are located in the bone marrow and a number of other tissues (13,14). Currently, bone marrow is the main source of MSCs. However, the aspiration of bone marrow involves invasive procedures and the yield of bone marrow-derived MSCs (BM-MSCs) decreases significantly with the age of the donor (15). The umbilical cord is an excellent alternative to bone marrow as a source of MSCs for experimental and clinical needs (16). However, data on the application of umbilical cord-derived MSCs is limited. In the present study, human umbilical cord-derived MSCs (hUC-MSCs) were used as stromal cells to evaluate their function in the drug resistance of T-ALL cells.

## Materials and methods

### Cell culture

The human T-ALL cell line, Jurkat, was cultured in suspension in RPMI 1640 medium (Gibco BRL, Grand Island, NY, USA) supplemented with 10% fetal bovine serum (FBS; Sijiqing, Hangzhou, China) and 1% penicillin/streptomycin (Gibco BRL). The cells were maintained at 37°C in a humidified chamber with 5% CO_2_ and routinely subcultured every 2–3 days, ensuring that the cell density in the culture did not exceed 1×10^6^ cells/ml.

hUC-MSC cultures were established from the umbilical cords of healthy donors using the direct plastic adherence method after informed consent had been obtained. The study was approved by the ethics committee of the School of Life Science and Biopharmaceutics of Guangdong Pharmaceutical University (Guangzhou, China). Briefly, the umbilical cord samples were sheared into 2–3-cm long segments and washed thoroughly to remove the residual cord blood. Each cord segment was dissected along its length to expose the blood vessels (two arteries and one vein), which were pulled away and discarded. The remaining cord tissue pieces were collected, minced into 1–2-mm^3^ fragments, plated separately in 6-cm polystyrene tissue culture dishes and maintained in DMEM/F12 medium (Gibco BRL) at 37°C in a humidified atmosphere with 5% CO_2_. The non-adherent tissues were removed on day seven and the culture medium was changed every 3–4 days thereafter. Approximately three weeks later, when well-developed colonies of fibroblast-like cells had appeared (80–90% confluent), the cultures were washed, harvested with 0.25% trypsin (Gibco BRL) and passed through a 100-μm sterile mesh to remove any residual tissue pieces. The filtered cells were then seeded in larger flasks for further expansion. The hUC-MSCs at passages 3–8, displaying a homogeneous mesenchymal immunophenotype and multipotent differentiation potential into adipocytic, osteoblastic and chondrocytic lineages, were used for the experiments.

### Polymerase chain reaction (PCR)

Total RNA was extracted from the hUC-MSCs and Jurkat cells using the TRIzol reagent (Invitrogen, Carlsbad, CA, USA) according to the manufacturer’s instructions. Reverse transcription was carried out using the PrimeScript II 1st strand cDNA synthesis kit (Takara, Otsu, Japan) with 1 μg total RNA as a template and oligo dT as a primer. All semiquantitative PCR experiments were performed using the same serially-diluted cDNA batches as templates. Amplification was performed at 95°C for 5 min followed by 38 cycles of 94°C for 30 sec, 56°C for 30 sec and 72°C for 30 sec, then a final extension at 72°C for 7 min. The amplified fragments were analyzed using electrophoresis on a 2% agarose gel. The gene-specific primers that were used for PCR are listed in Table I. The PCR of human β-actin was performed as a control.

### Detection of signaling molecules using flow cytometry

The Jurkat cells were harvested and prepared for flow cytometry following co-culture with hUC-MSCs. In brief, the hUC-MSCs were plated into 6-well plates at 2×10^5^ cells per well to form a confluent monolayer. Following this, 2×10^6^ Jurkat cells were added to each well of the adherent hUC-MSCs or cultured alone for 72 h. The co-cultured Jurkat cells were then separated from the hUC-MSCs by careful pipetting with ice-cold PBS. For the flow cytometry, the cells from the various cultures were washed and adjusted to a concentration of 5×10^6^ cells/ml in PBS. Aliquots of 100 μl cell suspension were then added into separate tubes. Fc receptors were blocked using the Fc Receptor Blocking reagent (Miltenyi Biotec, Bergisch Gladbach, Germany) for 15 min at 4°C. Surface antibodies were added and incubated for 30 min at 4°C in the dark. The unbound antibodies were removed by washing the cells twice in PBS and the cells were resuspended in 500 μl PBS for the final flow cytometric analysis on a Gallios cytometer (Beckman Coulter, Brea, CA, USA). The antibodies that were used were allophycocyanin (APC)-conjugated anti-CD45 (eBioscience, San Diego, CA, USA), carboxyfluorescein (CFS)-conjugated anti-Jagged1 (R&D systems, Minneapolis, MN, USA), phycoerythrin (PE)-conjugated anti-Notch1 (R&D systems), PE-conjugated anti-CD28 (eBioscience) and non-specific isotype-matched antibodies.

### Apoptosis analysis

To induce apoptosis, the Jurkat cells were cultured alone or co-cultured with hUC-MSCs for 72 h as described previously and then exposed to dexamethasone (Sigma, St Louis, MO, USA; final concentration 1 μM) for an additional 24 h. The blocking experiments were performed by incubating the hUC-MSCs and Jurkat cells with neutralizing monoclonal antibodies against human Jagged1 (R&D Systems; 1 μg/ml) prior to their inoculation into culture plates. Recombinant human Jagged1 proteins (R&D systems; 1 μg/ml) were used to stimulate the Jurkat cells directly. Apoptotic cell death was detected by Annexin V/propidium iodide (PI) staining using the MEBCYTO apoptosis kit (MBL, Nagoya, Japan). Briefly, the Jurkat cells from the various cultures were harvested, washed and immunolabeled with APC-conjugated anti-CD45. The cells were then washed and resuspended in 85 μl binding buffer, followed by incubation with 10 μl Annexin V-FITC and 5 μl PI at room temperature for 15 min in the dark. Following incubation, 400 μl binding buffer was added and the cell samples were measured using flow cytometry.

### Statistical analysis

All statistical calculations were performed using the GraphPad Prism software (GraphPad Software, Inc., La Jolla, CA, USA). The data are presented as the mean ± SD. When applicable, Student’s unpaired t-test, a one-way ANOVA and Holm-Sidak tests were used to determine significance. P<0.05 was considered to indicate a statistically significant difference.

## Results

### Characterization of hUC-MSCs

**Figure 1:**
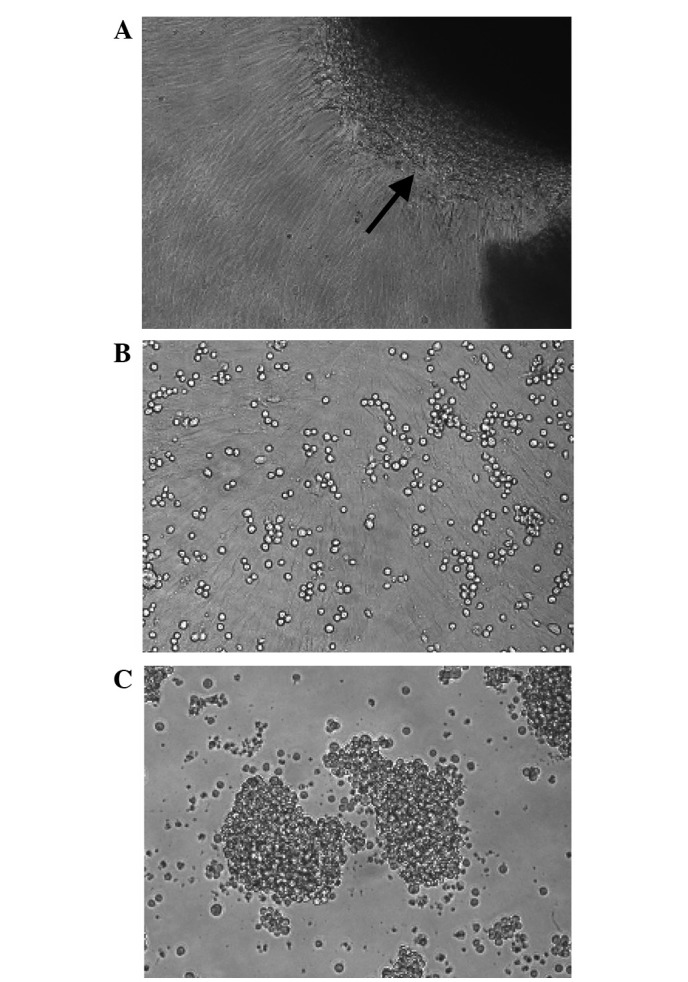
Morphological features of the hUC-MSCs and Jurkat cells. (A) hUC-MSCs in primary culture. The black arrow indicates a section of the adherent umbilical cord tissue fragment. (B) Jurkat cells grown on the confluent hUC-MSCs monolayer. (C) The Jurkat cells that were cultured alone observed under light microscopy at ×200 magnification. hUC-MSCs, human umbilical cord-derived mesenchymal stem cells.

**Figure 2:**
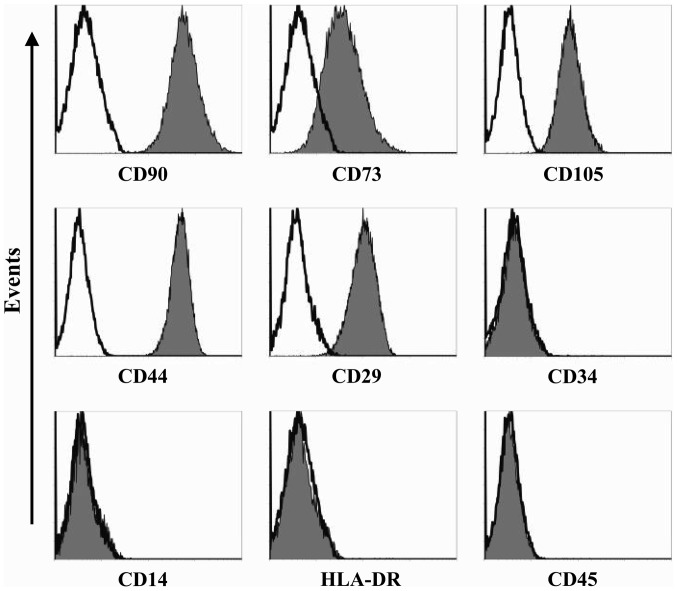
Aliquots of hUC-MSCs were incubated with each type of antibody associated with MSC immunophenotype separately and tested by flow cytometry. One representative analysis is reported. From left to right: CD90, CD73 and CD105 (upper row); CD44, CD29 and CD34 (middle row); and CD14, HLA-DR and CD45 (lower row). Open histograms, cells stained with isotype controls; filled histograms, cells labeled with specific antibodies. hUC-MSCs, human umbilical cord-derived mesenchymal stem cells; HLA-DR, human leukocyte antigen-DR.

**Figure 3:**
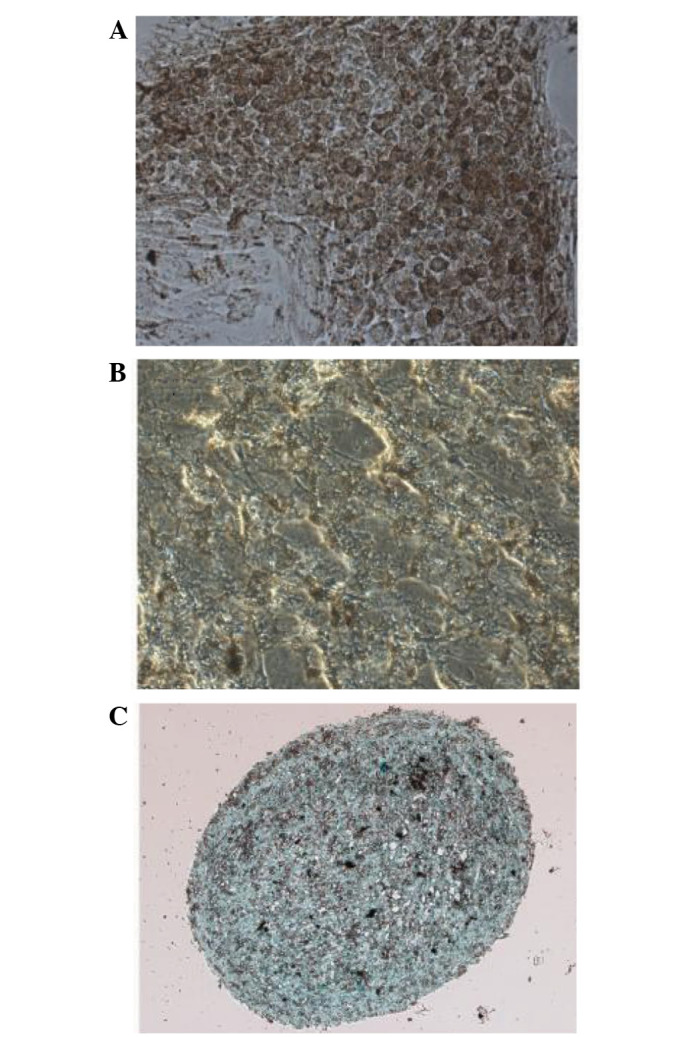
Multilineage differentiation capacity of hUC-MSCs. For (A) adipocytic and (B) osteoblastic differentiation, the hUC-MSCs were plated in 6-well plates at 2×10^4^ cells per well, treated with specific induction media and confirmed after a 3-week culture using (A) Oil Red O staining and (B) the calcium-cobalt sulfide method, respectively. (C) Chondrocytic differentiation was identified using Alcian blue staining following a 3-week culture with chondrocytic medium, which were added to a pellet of 2.5×10^5^ MSCs centrifuged at 150 × g for 5 min. hUC-MSCs, human umbilical cord-derived mesenchymal stem cells.

Fibroblast-like cells were successfully isolated from hUC tissues using the direct plastic adherence method in the present study (Fig. 1A). The cells formed whirlpool-like arrays when a confluent monolayer had developed (Fig. 1A and B). The flow cytometry analysis demonstrated that the hUC-MSCs showed good homogeneity and expressed MSC markers CD73, CD90, CD105, CD44 and CD29, but were negative for CD34, CD45, human leukocyte antigen (HLA)-DR and CD14 (Fig. 2). The same cells showed multilineage differentiation potential, as assessed by culturing in adipogenic, osteogenic or chondrogenic medium (Fig. 3).

### Expression of Notch ligands by hUC-MSCs

**Figure 4:**
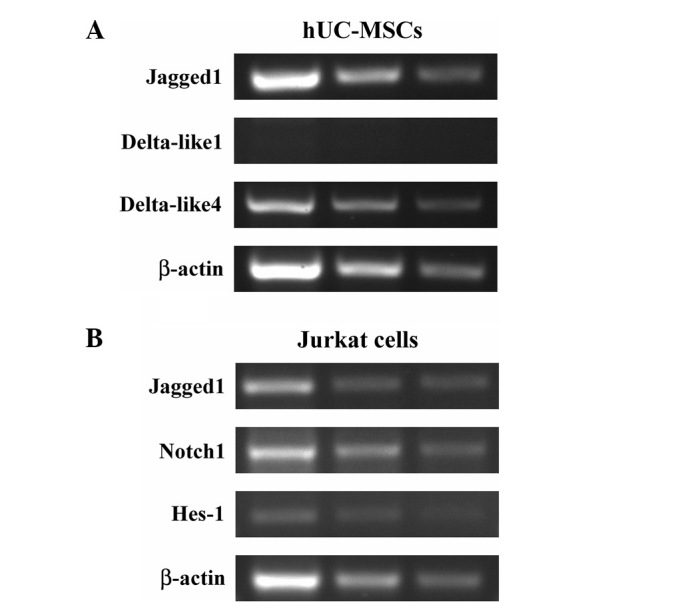
Expression of Notch-related genes in the hUC-MSCs and Jurkat cells. PCR was performed for the indicated transcripts from (A) hUC-MSCs and (B) Jurkat cells, respectively. Three serial dilutions of template cDNA are shown for each primer pair. hUC-MSCs, human umbilical cord-derived mesenchymal stem cells; Hes-1, hairy/enhancer-of-split 1; PCR, polymerase chain reaction.

To assess a possible role for the hUC-MSCs in inducing Notch signaling in the Jurkat T-ALL cells, the expression of Notch ligands Jagged1, DLL1 and DLL4 were examined in the hUC-MSCs by PCR using gene-specific primers, with β-actin as an internal control (Table I). This analysis revealed that transcripts for Jagged1 and DLL4 were detected in the hUC-MSCs, while the transcript for DLL1 was undetectable (Fig. 4A). In addition, Jagged1 was relatively highly expressed by the hUC-MSCs at the mRNA level.

### Upregulation of Notch1, Jagged1 and CD28 in Jurkat cells following contact with hUC-MSCs

**Figure 5:**
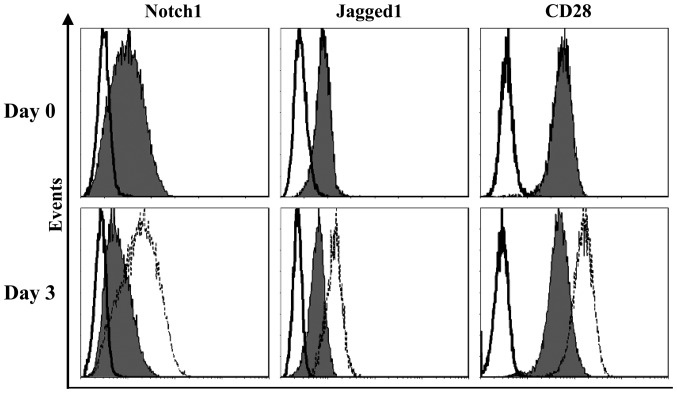
Representative flow cytometric measurements of Notch1, Jagged1 and CD28 expressed by Jurkat cells. Notch1, Jagged1 and CD28 expression in Jurkat cells was detected prior to (Day 0) and following (Day 3) a 3-day co-culture with hUC-MSCs. CD45^+^ Jurkat cells that were co-cultured with the hUC-MSCs (CD45^−^) were specifically gated and evaluated using a flow cytometry analysis. Open histograms with solid line, Jurkat cells stained with isotype controls; filled histograms, Jurkat cells cultured alone and labeled with specific mAbs; open histograms with dotted line, hUC-MSC-supported Jurkat cells labeled with specific mAbs. hUC-MSCs, human umbilical cord-derived mesenchymal stem cells; mAbs, monoclonal antibodies.

The expression of the Notch-related genes in the Jurkat cells was further analyzed. PCR analysis showed that the Jurkat cells expressed the Notch1 receptor and its ligand, Jagged1 (Fig. 4B), suggesting that the receptor and ligand pair may play a role in T-ALL cells. Hes-1, one of the main downstream molecules of the Notch pathway, was also expressed in the normally-cultured Jurkat cells (Fig. 4B), suggesting that Notch signaling is constitutively active in these cells. Flow cytometry was then used to assess the expression of Notch1, Jagged1 and CD28 in the Jurkat cells. As shown in Fig. 5, at basal conditions, the Jurkat cells expressed CD28 and moderate levels of Jagged1 and Notch1. Notably, following contact with the hUC-MSCs, an upregulation in the expression of all the molecules was observed in the Jurkat cells (Fig. 5; Table II), indicating their involvement in the functional interaction between the hUC-MSCs and the Jurkat T-ALL cell line.

### hUC-MSCs inhibit drug-induced apoptosis in Jurkat cells

**Figure 6:**
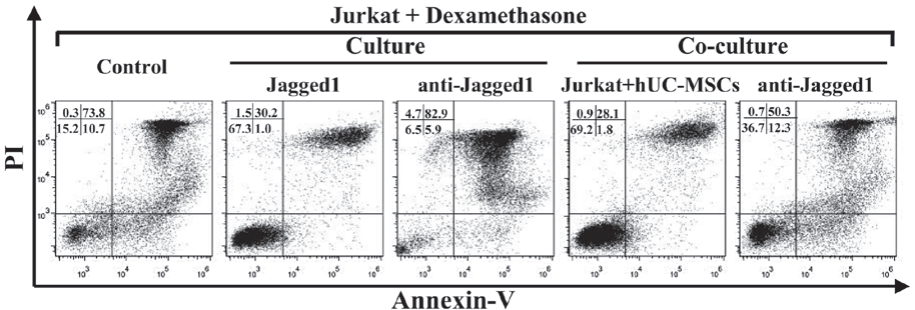
Typical flow cytometric analyses of dexamethasone-induced apoptosis in Jurkat cells under various culture conditions. Jurkat cells were cultured alone or co-cultured with hUC-MSCs under the indicated conditions for 72 h and treatment with dexamethasone for an additional 24 h. The percentage of live Jurkat cells were measured using Annexin V^−^/PI^−^ (bottom left quadrant) flow cytometry analysis following electronic gating on the CD45^+^ Jurkat cells. hUC-MSCs, human umbilical cord-derived mesenchymal stem cells; anti-Jagged1, neutralizing antibody against human Jagged1; Jagged1, recombinant human Jagged1 protein; PI, propidium iodide.

**Figure 7:**
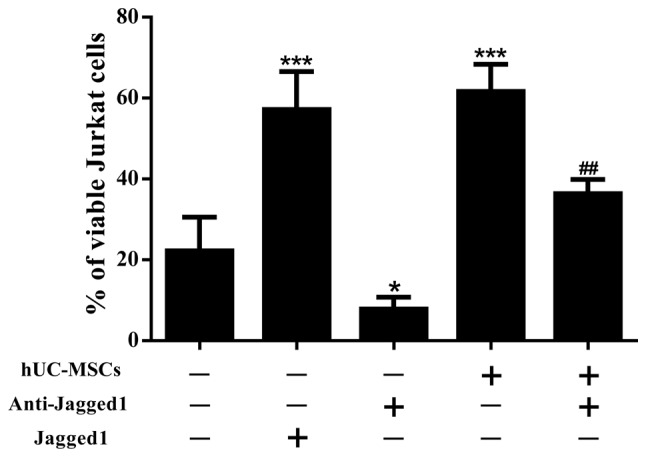
Percentages of live Jurkat cells exposed to dexamethasone under various culture conditions. The data in the bar graph represent the mean ± SD of three independent measurements. Statistical analysis was performed using a one-way ANOVA and Holm-Sidak test for multiple comparisons. ^*^P<0.05, vs. control Jurkat cells exposed to dexamethasone; ^***^P<0.001, vs. control Jurkat cells exposed to dexamethasone; ^##^P<0.01, vs. co-cultured Jurkat cells exposed to dexamethasone.

To study the capability of the hUC-MSCs to support leukemia cell survival, the Jurkat cells were cultured alone or co-cultured with the hUC-MSCs at a 10:1 ratio for 72 h and then exposed to dexamethasone for an additional 24 h. When observed using light microscopy, the Jurkat cells in the co-culture system showed an improved cell morphology compared with those that were cultured alone (Fig. 1B and C). As assessed by Annexin V/PI staining (Figs. 6 and 7), the Jurkat cells that were in contact with the hUC-MSCs underwent far less apoptosis induced by dexamethasone than those that were cultured alone. These data suggested that the hUC-MSCs were able to maintain the viability of the Jurkat T-ALL cells by preventing apoptosis.

### Jagged1 contributes to the drug resistance of Jurkat cells

To gain an improved understanding of the role of Jagged1 in the survival of the Jurkat cells, blocking experiments were performed using anti-Jagged1 neutralizing antibodies. By blocking Jagged1, a significant reduction in the percentage of live cells was achieved in the Jurkat cells that were exposed to dexamethasone, in the presence or absence of hUC-MSCs (Figs. 6 and 7). To further confirm the involvement of Jagged1 in the maintenance of Jurkat cell viability, recombinant Jagged1 was added to the Jurkat cell cultures. The exogenously-added Jagged1 significantly enhanced Jurkat cell survival in the presence of dexamethasone (Figs. 6 and 7). Overall, these results indicate that Jagged1 favored Jurkat cell survival under the pressure of drug treatment.

## Discussion

The interactions between hematological malignant cells and the elements of the stromal microenvironment play a key role in patient survival and the response to chemotherapy. BM-MSCs are commonly used as stromal cells for *in vitro* studies on hematological malignancies. Apart from being used as stromal cells for experimental requirements, MSCs also represent a homogeneous stem cell population with multilineage differentiation capabilities and immune regulatory properties (17), which make them an attractive tool for the cell-based therapy of numerous human disorders, including graft-versus-host disease (GvHD), in hematological malignancy patients undergoing hematopoietic stem cell transplantation (HSCT) (18,19). In the present study, hUC-MSCs were used as stromal cells. Compared with BM-MSCs, hUC-MSCs have several advantages, including an improved ability to expand, painless collection procedures, a lower risk of viral contamination and the fact that they are a possible source for autologous cell therapy (20). Despite these attractive features, the efficacy and safety of hUC-MSCs have to be evaluated in preclinical models prior to using them in clinical trials. In the co-culture experiments of the present study, the hUC-MSCs dramatically enhanced the *ex vivo* survival of the Jurkat T-ALL cells that were exposed to dexamethasone. This observation indicates a side-effect of the hUC-MSCs, which may maintain residual leukemia cells and lead to the recurrence of the disease. The same anti-apoptotic effects have also been observed on malignant cells in BM-MSCs (8,21), which constitutes a significant limitation for their clinical application and may explain to a certain extent the emerging evidence indicating that the co-transplantation of MSCs may increase the risk of hematological malignancy relapse following HSCT (22).

To explore the underlying mechanism, the present study focused on Notch signaling due to its involvement in the pathogenesis of T-ALL and its potential role in regulating cell apoptosis. The interaction between Notch receptors and the membrane-bound ligands of the Delta and Jagged families is critical for the activation of Notch signaling (6). Mammals have four Notch receptors (Notch1–4) that bind to five various transmembrane ligands, DLL1, 3 and 4 and Jagged1 and 2 (6). The actions of the ligands differ in the initiation of Notch signaling. Jagged1 and 2 and DLL1, commonly known as Delta/Serrate/LAG-2 (DSL), are ligands for Notch receptors 1–4 (6,23). DLL4 is able to bind and activate the Notch1 and 4 receptors (6,23, 24), whereas DLL3 is able to bind and activate Notch1 or similar Notch receptors (6,23,25). Furthermore, Notch signaling that is triggered by various ligands may result in a diverse or opposed biological outcome (12).

In the present study, one of the Notch ligands, Jagged1, was observed to be expressed by the hUC-MSCs and the Jurkat T-ALL cell line. Jagged1 is a membrane-spanning protein with a large extracellular domain that is important for Notch receptor binding (26). This ligand has been indicated to be expressed at a significant level in BM-MSCs (27) and is associated with certain BM-MSC functions, including the regulation of the hematopoietic stem cell (HSC) niche (28), suppressive effects on immune cells (29) and cellular differentiation (30). The expression of Jagged1 by hUC-MSCs may initiate the stimulation of Notch signaling in the Jurkat cells by binding to the Notch1 receptor and thus, may contribute to the hUC-MSC-induced survival of the T-ALL cells. By contrast, Jagged1 was also expressed by the Jurkat T-ALL cell line, in addition to the constitutive expression of the Notch1 receptor. The contemporary expression of the Notch1 receptor and its ligand on the cell surface may lead to auto- or reciprocal activation of Notch signaling among the T-ALL cells and thus, favor their own survival. As expected, in the present study, the blockade of Jagged1 significantly abrogated the drug resistance of the Jurkat cells that were in contact with the hUC-MSCs, and also increased Jurkat cell sensitivity to dexamethasone in the absence of the hUC-MSCs. By contrast, the addition of recombinant Jagged1 protein enhanced the survival of the Jurkat cells that were treated with dexamethasone. The results of the blocking and stimulating experiments implied that Jagged1 contributed to hUC-MSC-induced drug resistance and to the self-maintenance of the Jurkat T-ALL cells.

In order to identify certain targets that are involved in the prevention of apoptosis mediated by hUC-MSCs, CD28 expression was assessed in the Jurkat cells in the present study. CD28 is one of the co-stimulatory molecules that are expressed by T cells (31). In the present study, the high expression level of CD28 in the Jurkat T-ALL cell line was more apparent following contact with the hUC-MSCs. CD28 has been identified as a direct target of Notch signaling (32), and has also been shown to be associated with the enhanced survival of immature (33) and activated (34) T cells. Therefore, the role of CD28 in the drug resistance of T-ALL warrants further investigation.

In conclusion, the present data indicate that the hUC-MSCs induced the drug resistance of the Jurkat T-ALL cell line. Jagged1, one of the Notch ligands, contributes to this phenomenon, which may also play a role in the self-maintenance of T-ALL cells and thus be a potential target for the treatment of human T-ALL. The evaluation of additional T-ALL cell lines, as well as primary T-ALL cells, using this co-culture system is necessary to expand these observations and to lay a theoretical basis for the development of new therapeutic strategies for T-ALL in the future.

## Figures and Tables

**Table I:** Primers for the PCR analysis.

Genes	Primers, 5′-3′	Size of targets, bp
Notch1		
F	CTACCTGTCAGACGTGGCCT	357
R	CGCAGAGGGTTGTATTGGTT	
Jagged1		
F	CTCATCAGCCGTGTCTCAAC	297
R	GGCACACACACTTAAATCCG	
DLL1		
F	TATCCGCTATCCAGGCTGTC	297
R	GGTGGGCAGGTACAGGAGTA	
DLL4		
F	AAGGCTGCGCTACTCTTACC	538
R	ATCCTCCTGGTCCTTACAGC	
Hes-1		
F	ATCACACAGGATCCGGAGCT	300
R	TGACACTGGCTGGGGTAGC	
β-actin		
F	CTACAATGAGCTGCGTGTGG	314
R	CGGTGAGGATCTTCATGAGG	

PCR, polymerase chain reaction; F, forward primer; R, reverse primer; DLL, Delta-like; Hes-1, hairy/enhancer-of-split 1.

**Table II:** Expression of Notch-related molecules by Jurkat cells cultured alone or co-cultured with hUC-MSCs.

	Jurkat cells		
	
	Jagged1	Notch1	CD28
Alone	0.6±0.2	1.2±0.5	5.7±2.0
Co-culture	1.6±0.4	2.6±0.7	17.6±3.5
Student’s t-test	P<0.05	P<0.05	P<0.01

The results are expressed as the mean ± SD of the MFI values (n=3) of the Jurkat cells that were cultured alone or co-cultured with hUC-MSCs for 72 h. hUC-MSCs, human umbilical cord-derived mesenchymal stem cells; MFI, mean fluorescence intensity.
